# STUB1 is acetylated by KAT5 and alleviates myocardial ischemia-reperfusion injury through LATS2-YAP-β-catenin axis

**DOI:** 10.1038/s42003-024-06086-9

**Published:** 2024-04-01

**Authors:** Can Liu, Zhongxuan Gui, Cheng An, Fei Sun, Xiaotian Gao, Shenglin Ge

**Affiliations:** Department of Cardiovascular Surgery, The First Affiliated Hospital of Anhui Medical University, Hefei, 230022 Anhui Province P.R. China

**Keywords:** Cell biology, Cardiovascular diseases

## Abstract

Myocardial ischemia-reperfusion injury (MIRI) is involved in the pathogenesis of multiple cardiovascular diseases. This study elucidated the biological function of lysine acetyltransferase 5 (KAT5) in cardiomyocyte pyroptosis during MIRI. Oxygen-glucose deprivation/reoxygenation and left anterior descending coronary artery ligation were used to establish MIRI models. Here we show, KAT5 and STIP1 homology and U-box-containing protein 1 (STUB1) were downregulated, while large tumor suppressor kinase 2 (LATS2) was upregulated in MIRI models. KAT5/STUB1 overexpression or LATS2 silencing repressed cardiomyocyte pyroptosis. Mechanistically, KAT5 promoted STUB1 transcription via acetylation modulation, and subsequently caused ubiquitination and degradation of LATS2, which activated YAP/β-catenin pathway. Notably, the inhibitory effect of STUB1 overexpression on cardiomyocyte pyroptosis was abolished by LATS2 overexpression or KAT5 depletion. Our findings suggest that KAT5 overexpression inhibits NLRP3-mediated cardiomyocyte pyroptosis to relieve MIRI through modulation of STUB1/LATS2/YAP/β-catenin axis, providing a potential therapeutic target for MIRI.

## Abstract (web summary)

In this study, the authors show that lysine acetyltransferase 5 inhibits NLRP3-mediated cardiomyocyte pyroptosis via STUB1, relieving myocardial ischemia-reperfusion injury.

## Introduction

Blood flow reperfusion contributes to the improvement of ischemic myocardial function; however, this process may result in functional and structural damage, cardiac arrhythmias, and metabolic defects in the heart, which is termed myocardial ischemia-reperfusion injury (MIRI)^1,2^. MIRI may cause serious consequences, such as myocardial cell death and myocardial infarction, thereby threatening the life and health of patients^3^. It has been recognized that the pathogenesis of MIRI is complex^4^. Thus, it is crucial to uncover the complicated pathological mechanisms and develop efficient therapies to mitigate MIRI.

A growing body of evidence indicated that MIRI is associated with the induction of pyroptosis^5^. Pyroptosis is considered a key manifestation of inflammation in response to MIRI^6^. As an inflammatory programmed cell death, pyroptosis can be triggered by NOD-like receptor thermal protein domain associated protein 3 (NLRP3) inflammasome comprised of NLRP3, ASC, and caspase-1 proteins^7^. The activation of NLRP3 inflammasome can exacerbate MIRI via promoting caspase-1-mediated pyroptosis and subsequent release of pro-inflammatory cytokines IL-1β and IL-18^8^. It has been reported that suppression of NLRP3 inflammasome-mediated pyroptosis conferred protection against MIRI and improved the cardiac function of rats^9^.

Lysine acetyltransferase 5 (KAT5), also known as TIP60, is a histone acetyltransferase that is responsible for transcriptional regulation^10^. Hishikawa and colleagues found that KAT5 could attenuate kidney I/R injury via transcriptional regulation of KCC3^11^. KAT5 deficiency led to cardiomyocyte death and cardiac dysfunction in mice, suggesting the crucial influence of KAT5 in heart function^12^. However, whether KAT5 can modulate MIRI has not been reported. STIP1 homology and U-box-containing protein 1 (STUB1) is an E3-ligase that facilitates protein degradation^13^. STUB1 is widely expressed in various tissues, including the heart. Ranek et al. documented that the enhanced phosphorylation and expression of STUB1/CHIP protected against MIRI and improved heart dysfunction in mice^14^. Bioinformatics analysis predicted that KAT5 might bind to STUB1 promoter. Therefore, we speculated that KAT5 might modulate STUB1 transcription via histone acetylation modulation of STUB1.

Hippo-YAP pathway activation exerts crucial roles in myocardial regeneration, which represents as an effective therapeutic strategy for MIRI^15^. Furthermore, Ube2s-mediated stabilization of β-catenin was proved to ameliorate MIRI in mice through activation of HIF-1α^16^. Li et al. documented that the nuclear interaction between YAP and β-catenin inactivated NLRP3, thereby repressing liver inflammation in liver injury^17^. Large tumor suppressor kinase 2 (LATS2) is a cascade kinase of the Hippo pathway that can phosphorylate YAP to result in its inactivation^18^. Activation of LATS2 was documented to lead to heart failure via triggering p53-dependent apoptosis of cardiomyocytes^18^. Interestingly, bioinformatics analysis predicted that STUB1 possessed ubiquitination sites on LATS2. In this context, we speculated that KAT5-mediated transcription of STUB1 via histone acetylation might result in ubiquitination and degradation of LATS2 and then repressed NLRP3 inflammasome-mediated pyroptosis via activating YAP/β-catenin pathway, thereby attenuating MIRI. In this study, we aimed to verify this speculation and further uncovered the complicated pathogenesis of MIRI.

## Results

### OGD/R triggers NLRP3-mediated pyroptosis and dysregulation of LATS2, YAP, and β-catenin in vitro

**Fig. 1:**
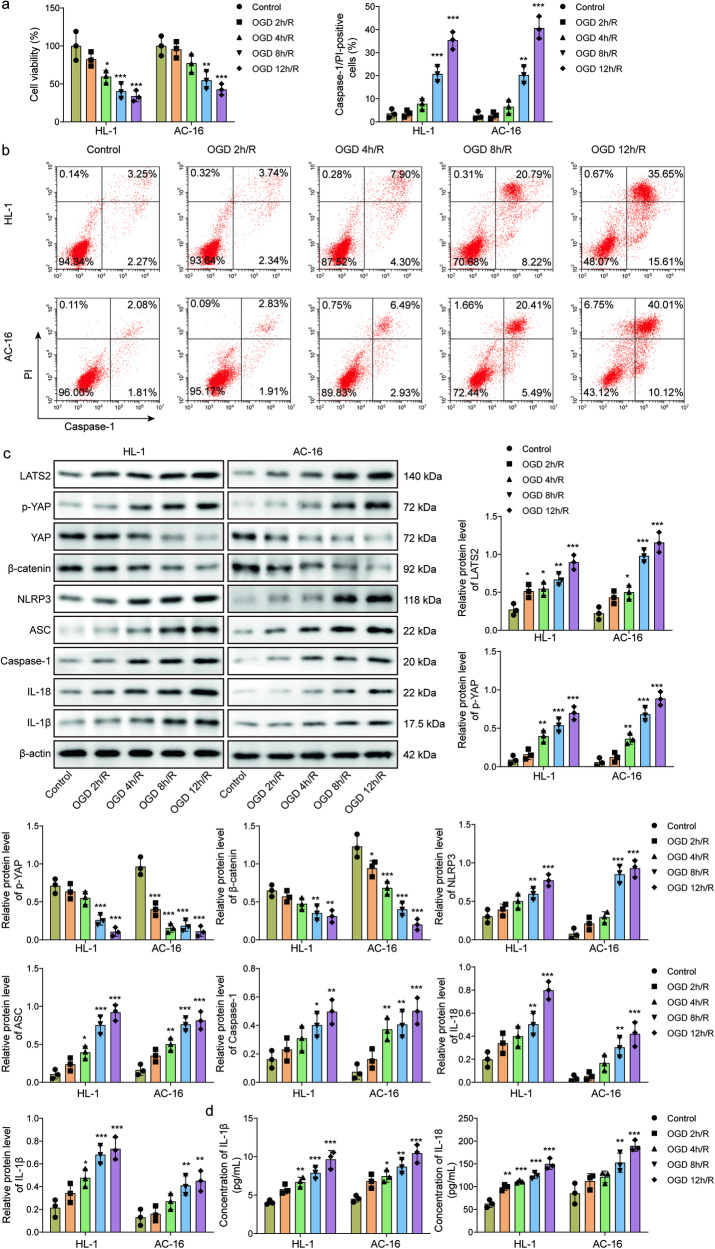
NLRP3-mediated pyroptosis induction and dysregulation of LATS2, YAP, and β-catenin in OGD/R-stimulated cardiomyocytes in vitro*.* AC16 and HL-1 cells were subjected to oxygen-glucose deprivation (OGD) for 2, 4, 8, or 12 h, followed by reoxygenation for 6 h. **a** AC16 and HL-1 cell viability was determined by CCK-8. **b** Cell pyroptosis was evaluated by flow cytometry. **c** Western blotting analysis of LATS2, p-YAP, YAP, β-catenin, NLRP3, ASC, Caspase-1, IL-18, IL-1β protein levels. **d** IL-18 and IL-1β release was determined by ELISA. Data represent the mean ± SD. *n* = 3 independent experiments. One-way ANOVA followed by Bonferroni was performed for statistical analysis in a-d. Box plots represent median with minimum and maximum whiskers. *p < 0.05, **p < 0.01, ***p < 0.001 versus control group.

First, cardiomyocytes were exposed to OGD/R to simulate MIRI in vitro. Cardiomyocyte viability was gradually declined after OGD/R challenge (Fig. 1a). In addition, flow cytometry data demonstrated that pyroptosis percentage of OGD/R-exposed cardiomyocytes (Caspase-1/PI double-positive cells) was time-dependently increased (Fig. 1b). Moreover, LATS2, p-YAP, NLRP3, ASC, Caspase-1, IL-18, IL-1β were upregulated, while YAP and β-catenin were downregulated by OGD/R stimulation in a time-dependent manner (Fig. 1c). Accordingly, IL-18 and IL-1β release from OGD/R-exposed cardiomyocytes was reinforced with the extension of OGD/R time (Fig. 1d). These findings suggested that dysregulation of LATS2, YAP, and β-catenin might participate in the progression of MIRI.

### LATS2 aggravates MIRI in vitro via inactivation of YAP/β-catenin pathway

**Fig. 2:**
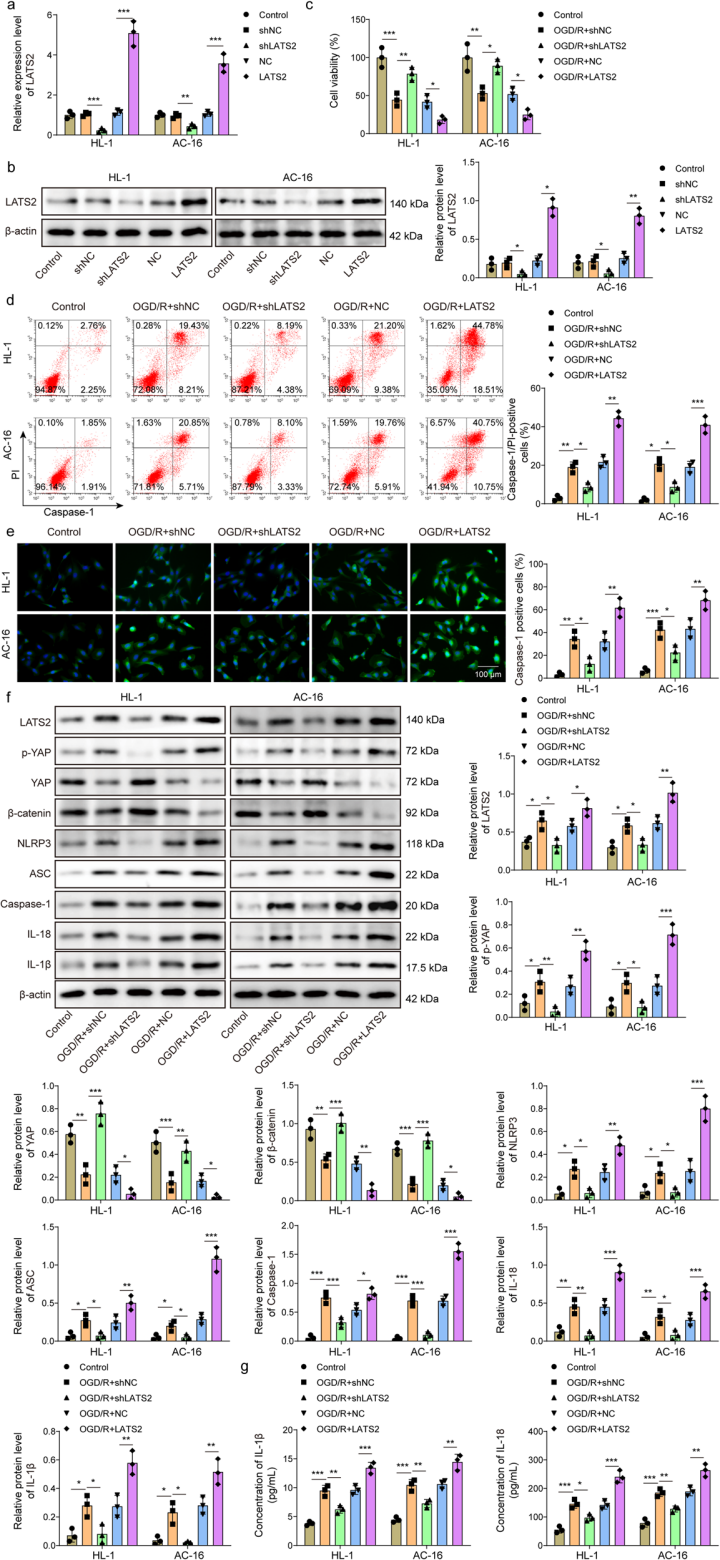
MIRI is aggravated by LATS2 via inactivation of YAP/β-catenin pathway in vitro. AC16 and HL-1 cells were transfected with sh-LATS2 or LATS2 overexpression lentivirus. **a**, **b** LATS2 expression in AC16 and HL-1 cells was assessed by RT-qPCR and Western blotting, respectively. AC16 and HL-1 cells were transfected with sh-LATS2 or LATS2 overexpression lentivirus, and then were stimulated with OGD/R. **c** Cell viability was detected by CCK-8. **d** The pyroptosis of cells was measured by flow cytometry. **e** Caspase-1 expression in AC16 and HL-1 cells was observed by immunofluorescence staining (green fluorescence) Scale bar = 100 μm. **f** The protein levels of LATS2, p-YAP, YAP, β-catenin, NLRP3, ASC, Caspase-1, IL-18, IL-1β expression in AC16 and HL-1 cells were determined by western blotting. **g** IL-18 and IL-1β production was detected by ELISA. Data represent the mean ± SD. *n* = 3 independent experiments. One-way ANOVA followed by Bonferroni was performed for statistical analysis in **a**–**g**. Box plots represent median with minimum and maximum whiskers. *p < 0.05, **p < 0.01, ***p < 0.001 versus indicated group.

To determine whether LATS2 could affect MIRI in vitro, cardiomyocytes were transfected with sh-LATS2 or LATS2 overexpression plasmid. The overexpression and silencing efficiency of LATS2 in AC16 and HL-1 cells was validated (Fig. 2a, b). LATS2 depletion enhanced OGD/R-exposed cardiomyocyte viability, whereas LATS2 overexpression exerted the opposite role (Fig. 2c). Additionally, OGD/R-induced pyroptosis of AC16 and HL-1 cells was weakened by LATS2 silencing, but intensified by LATS2 overexpression (Fig. 2d). The increased expression of Caspase-1 induced by OGD/R could be reversed in LATS2-depleted cells, while further enhanced in LATS2-overexpressed cells (Fig. 2e). Furthermore, LATS2 deficiency reversed OGD/R-mediated upregulation of LATS2, p-YAP, NLRP3, ASC, Caspase-1, IL-18, IL-1β, and downregulation of YAP and β-catenin in cardiomyocytes. However, we observed opposite results in LATS2-overexpressed group (Fig. 2f). In addition, IL-18 and IL-1β release from OGD/R-exposed cells could be repressed by LATS2 downregulation, but further promoted by LATS2 upregulation (Fig. 2g). Collectively, these results proved that LATS2 promoted NLRP3-mediated pyroptosis during MIRI via inactivating YAP/β-catenin pathway.

### STUB1 leads to ubiquitination and degradation of LATS2

**Fig. 3:**
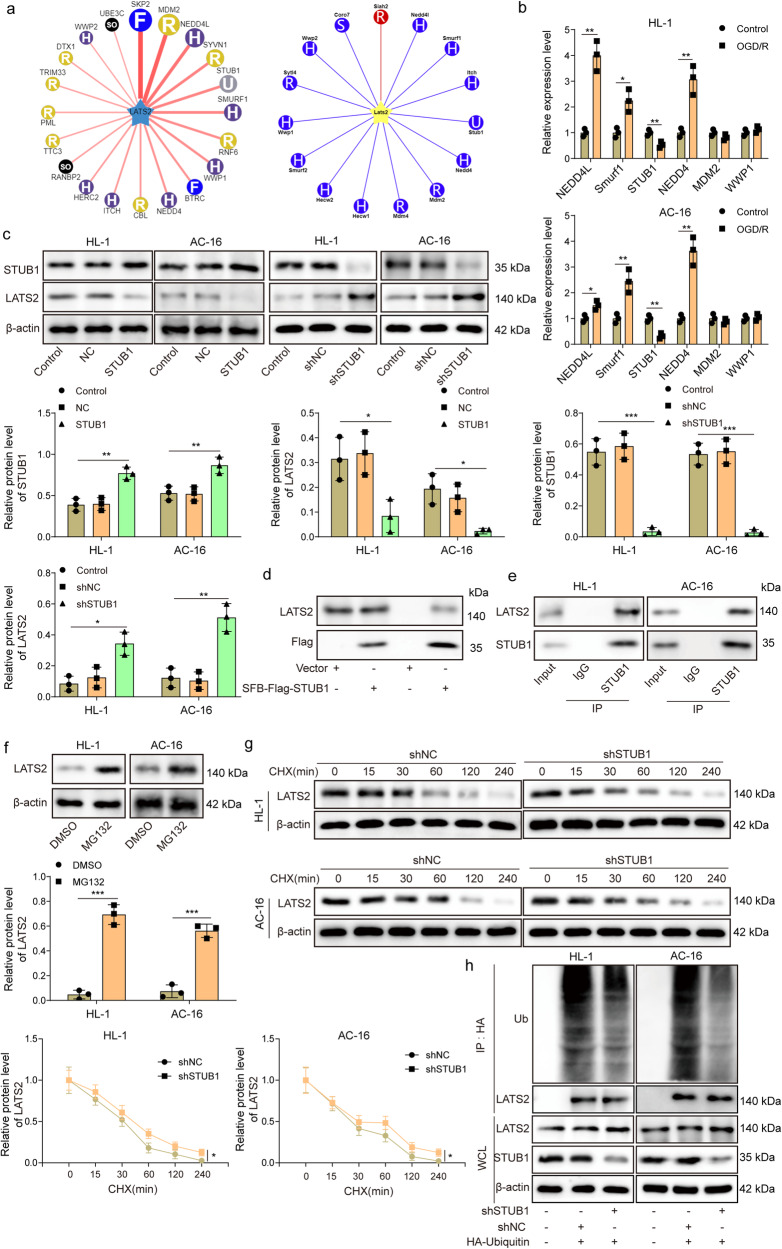
STUB1 leads to ubiquitination and degradation of LATS2. **a** Ubibrowser datebase predicted LATS2 as a target of multiple E3 ubiquitin ligases. **b** AC16 and HL-1 cells were exposed to OGD/R, and RT-qPCR was performed to detect E3 ubiquitin ligases *NEDD4L*, *Smurf1*, *STUB1, NEDD4, MDM2*, and *WWP1* expression in AC16 and HL-1 cells. **c** AC16 and HL-1 cells were transfected with sh-STUB1, and STUB1 overexpression lentivirus, and then STUB1 and LATS2 protein levels were assessed by western blotting. **d** 293 T cells were transfected with vector or SFB-Flag-STUB1 plasmid for 48 h, and the interaction between exogenous STUB1 and LATS2 proteins was evaluated by Co-IP assay. **e** The interaction between endogenous STUB1 and LATS2 proteins was confirmed by Co-IP assay. **f** After treatment with 100 nM MG132 or DMSO for 48 h, LATS2 protein level was detected by western blotting. **g** AC16 and HL-1 cells transfected with sh-NC or sh-STUB1 were treated with 50 μg/mL cycloheximide (CHX) for 0, 15, 30, 60, 120, 240 min, and then LATS2 level was detected by western blotting. **h** AC16 and HL-1 cells were transfected with sh-STUB1 or sh-NC, together with HA-Ubiquitin for 48 h, the ubiquitination and degradation of LATS2 was measured by Co-IP assay. Data represent the mean ± SD. *n* = 3 independent experiments. **b**, **f**, **g** Student’s *t* test was used for statistical analysis. **c** One-way ANOVA followed by Bonferroni was performed for statistical analysis. Box plots represent the median with minimum and maximum whiskers. *p < 0.05, **p < 0.01, ***p < 0.001 versus indicated group.

Next, we sought to investigate the upstream regulatory mechanism of LATS2 in MIRI. Ubibrowser analysis predicted that LATS2 might be ubiquitinated by a series of E3 ubiquitin ligases, such as NEDD4L, Smurf1, STUB1, NEDD4, MDM2, and WWP1 (Fig. 3a). RT-qPCR assay indicated that only *STUB1* was downregulated in OGD/R-stimulated AC16 and HL-1 cells (Fig. 3b). Therefore, STUB1 was focused on in the subsequent experiments. After transfection with sh-STUB1, the protein level of STUB1 was decreased, but LATS2 level was increased in AC16 and HL-1 cells, while STUB1 overexpression presented the opposite trend (Fig. 3c). However, the mRNA level of *LATS2* was not changed after OGD/R stimulation or STUB1 overexpression (Supplementary Fig. 1a, b). Co-IP assay validated the exogenous and endogenous interaction between STUB1 and LATS2 proteins (Fig. 3d, e). After treatment with MG132, an inhibitor of proteasome, LATS2 protein level was strikingly enhanced (Fig. 3f). Besides, the degradation of LATS2 protein in cycloheximide (CHX)-treated AC16 and HL-1 cells was delayed by STUB1 silencing (Fig. 3g). Furthermore, STUB1 silencing could reduce ubiquitination and degradation of LATS2 protein (Fig. 3h). Taken together, STUB1 repressed LATS2 protein expression via ubiquitination and degradation of LATS2.

### STUB1 attenuates MIRI in vitro through modulating LATS2/YAP/β-catenin axis

**Fig. 4:**
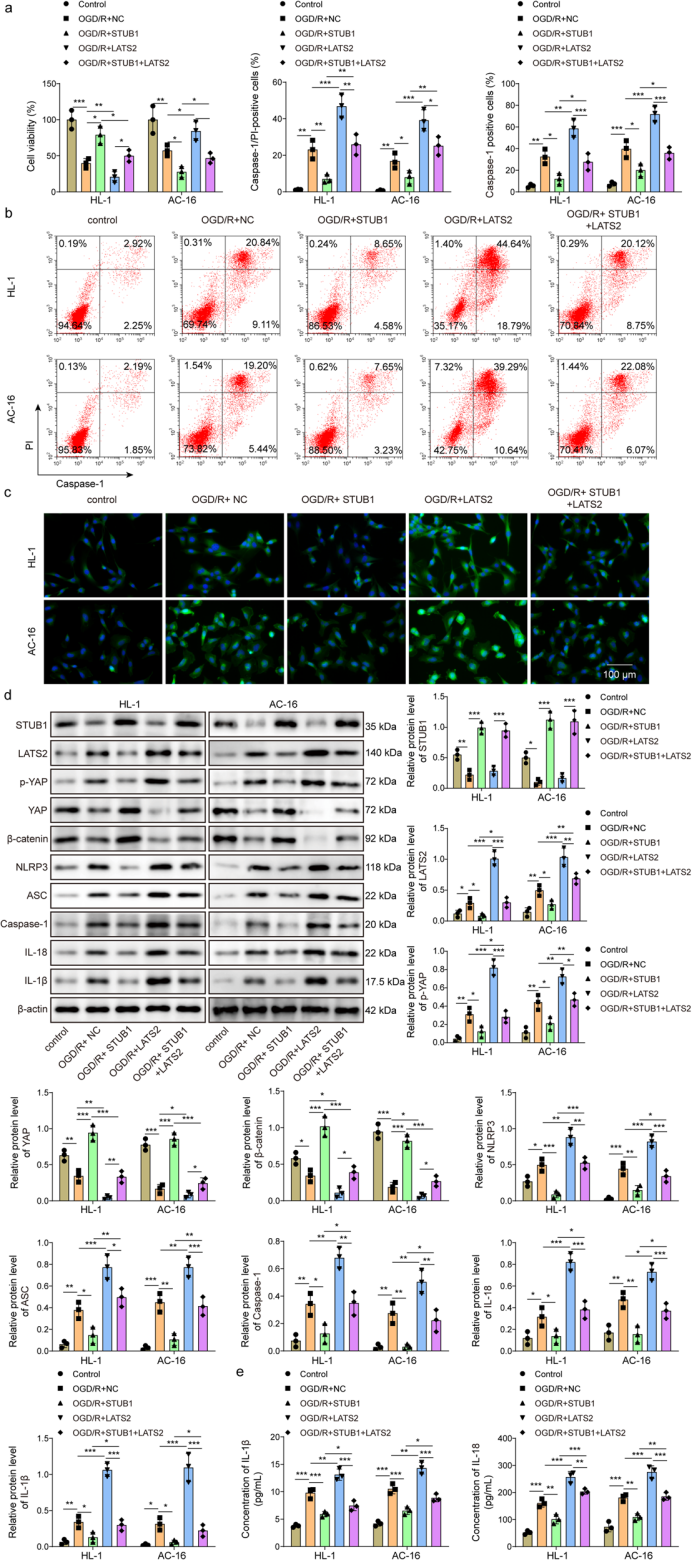
STUB1 attenuates MIRI in vitro through modulating LATS2/YAP/β-catenin axis. AC16 and HL-1 cells were transfected with STUB1, LATS2 overexpression lentivirus, or a combination of them, followed by exposure to OGD/R. **a** Cell viability was measured by CCK-8. **b** Flow cytometry was utilized to determine cell pyroptosis (scale bar = 100 μm). **c** Caspase-1 expression was detected by immunofluorescence staining (green fluorescence). Scale bar = 100 μm. **d** STUB1, LATS2, p-YAP, YAP, β-catenin, NLRP3, ASC, Caspase-1, IL-18, IL-1β levels in AC16 and HL-1 cells were evaluated by western blotting. **e** IL-18 and IL-1β release from AC16 and HL-1 cells was assessed by ELISA. Data represent the mean ± SD. *n* = 3 independent experiments. One-way ANOVA followed by Bonferroni was performed for statistical analysis in **a**–**e**. Box plots represent median with minimum and maximum whiskers. *p < 0.05, **p < 0.01, ***p < 0.001 versus indicated group.

To examine whether STUB1 could affect MIRI progression through regulation of LATS2, we transfected overexpression lentivirus for STUB1 or LATS2, or a combination of them into AC16 and HL-1 cells. As detected by CCK-8, overexpression of STUB1 raised the viability of OGD/R-stimulated cardiomyocytes, whereas STUB1 overexpression-mediated elevation in cell viability was reversed by LATS2 overexpression (Fig. 4a). Moreover, the pyroptosis percentage after OGD/R exposure was reduced by STUB1 overexpression, which was abolished by co-transfection with LATS2 overexpression lentivirus (Fig. 4b). Besides, the percentage of Caspase-1 positive cells after exposure to OGD/R was remarkably decreased in STUB1-overexpressed cells, however, co-overexpression of LATS2 counteracted this change (Fig. 4c). Western blotting results showed that overexpression of STUB1 reduced LATS2, p-YAP, NLRP3, ASC, Caspase-1, IL-18, IL-1β levels, and enhanced YAP and β-catenin levels in OGD/R-exposed cardiomyocytes, whereas these changes could be abrogated by LATS2 overexpression (Fig. 4d). Accordingly, OGD/R-induced release of IL-18 and IL-1β was inhibited by STUB1 overexpression, and this effect mediated by STUB1 overexpression was counteracted after LATS2 was overexpressed (Fig. 4e). The above results suggested that STUB1 repressed LATS2 expression to attenuate OGD/R-induced pyroptosis in cardiomyocytes.

### KAT5 protects against NLRP3-mediated pyroptosis in the in vitro model of MIRI

**Fig. 5:**
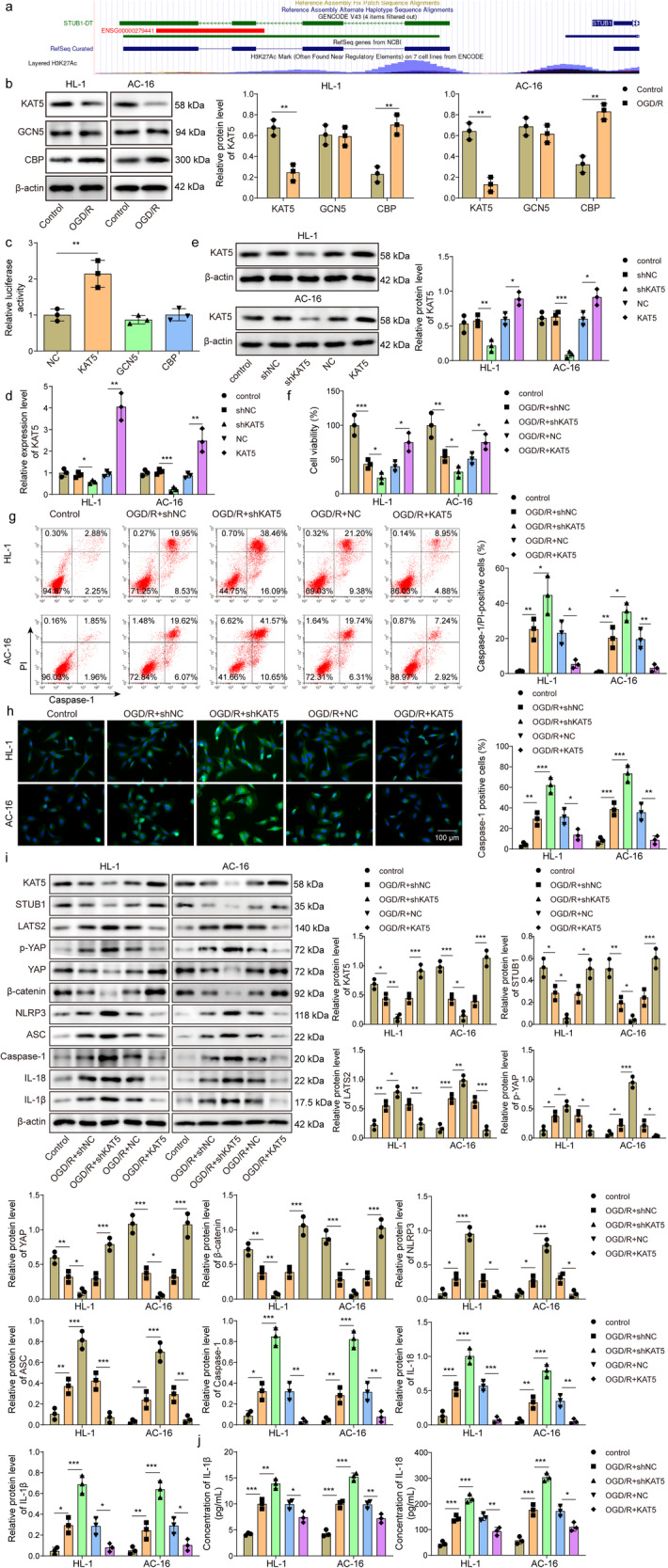
KAT5 attenuates apoptosis and NLRP3-mediated pyroptosis during MIRI in vitro. **a** UCSC database predicted the enrichment of H3K27ac to *STUB1* promoter. **b** AC16 and HL-1 cells were stimulated with OGD/R, and KAT5, GCN5, and CBP protein levels were detected by western blotting analysis. **c** AC16 and HL-1 cells were transected with KAT5, GCN5, and CBP overexpression vector for 48 h, and the direct binding of KAT5, GCN5, and CBP to *STUB1* promoter was evaluated by dual-luciferase reporter assay. **d**, **e** AC16 and HL-1 cells were transfected with shKAT5 or KAT5 overexpression lentivirus, and KAT5 expression was assessed by RT-qPCR and western blotting, respectively. AC16 and HL-1 cells were transfected with shKAT5 or KAT5 overexpression lentivirus, followed by exposure to OGD/R. **f** Cell viability was measured by CCK-8. **g** Flow cytometry was adopted to measure cell pyroptosis. **h** Caspase-1 expression was detected by immunofluorescence staining (green fluorescence). Scale bar = 100 μm. **i** KAT5, STUB1, LATS2, YAP, p-YAP, β-catenin, NLRP3, ASC, Caspase-1, IL-18, IL-1β levels were measured by western blotting. **j** IL-18 and IL-1β release was detected by ELISA. Data represent the mean ± SD. *n* = 3 independent experiments. **b**, **c** Student’s *t* test was used for statistical analysis. **d**–**j** One-way ANOVA followed by Bonferroni was performed for statistical analysis. Box plots represent the median with minimum and maximum whiskers. *p < 0.05, **p < 0.01, ***p < 0.001 versus indicated group.

It has been reported that histone modification exerts a pivotal role in the transcriptional regulation of target genes^19^. Accordingly, we found that histone marker H3K27ac was highly enriched at STUB1 promoter (Fig. 5a). In addition, we detected the levels of several histone acetyltransferases, including KAT5, GCN5, and CBP after OGD/R stimulation. We found that GCN5 level was not affected, and KAT5 was downregulated, while CBP was upregulated in OGD/R-exposed cells (Fig. 5b). Dual-luciferase reporter assay showed that the luciferase activity of *STUB1* promoter was enhanced by KAT5 overexpression, but not influenced by GCN5 or CBP overexpression (Fig. 5c). Thus, KAT5 acted as an upstream modulator of STUB1, which was selected in the subsequent experiments. Next, sh-KAT5 or KAT5 overexpression lentivirus was transfected into AC16 and HL-1 cells to evaluate the role of KAT5 in MIRI. As assessed by RT-qPCR and Western blotting, the silencing or overexpression efficiency of KAT5 was confirmed (Fig. 5d, e). KAT5 overexpression enhanced viability and restrained pyroptosis of OGD/R-stimulated cells, whereas KAT5 knockdown exhibited the opposite effects (Fig. 5f, g). In addition, OGD/R-mediated the enhanced expression of Caspase-1, LATS2, p-YAP, NLRP3, ASC, IL-18, IL-1β, and reduced expression of KAT5, STUB1, YAP, β-catenin could be reversed by KAT5 overexpression, but intensified by KAT5 deficiency (Fig. 5h, i). As expected, KAT5 overexpression repressed the release of IL-18 and IL-1β after OGD/R exposure, whereas KAT5 knockdown resulted in the opposite results (Fig. 5j). Thus, KAT5 exerted protection against MIRI via repressing NLRP3-mediated pyroptosis.

### KAT5 acetylates STUB1 to relieve OGD/R-induced cardiomyocyte injury via LATS2/YAP/β-catenin axis

**Fig. 6:**
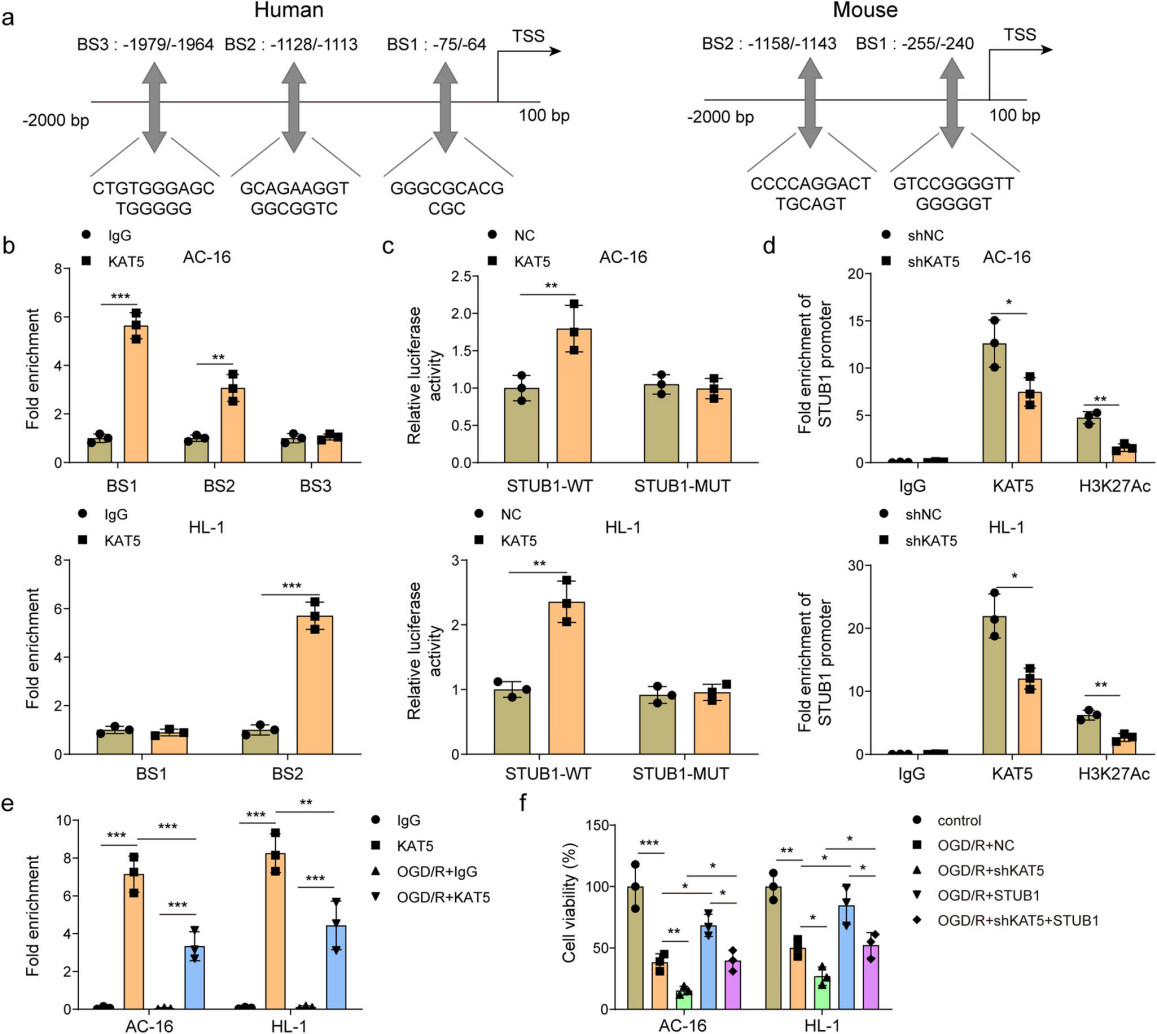
KAT5 acetylates STUB1 to relieve OGD/R-induced cardiomyocyte injury via LATS2/YAP/β-catenin axis. **a** Bioinformatic analysis predicted the potential binding sites (BS) of KAT5 to *STUB1* promoter. The direct binding of KAT5 to *STUB1* promoter was validated by ChIP (**b**) and dual-luciferase reporter assay (**c**). **d** The direct binding of KAT5/H3K27ac to *STUB1* promoter in KAT5-silenced AC16 and HL-1 cells was detected by ChIP assay. **e** ChIP-re-ChIP assay validated the interaction of KAT5 with H3K27ac on the *STUB1* promoter. AC16 and HL-1 cells were transfected with shKAT5, STUB1 overexpression lentivirus, or a combination of them, followed by stimulation with OGD/R. **f** Cell viability was detected by CCK-8. Data represent the mean ± SD. *n* = 3 independent experiments. **b**–**d** Student’s *t* test was used for statistical analysis. **e**, **f** One-way ANOVA followed by Bonferroni was performed for statistical analysis. Box plots represent median with minimum and maximum whiskers. *p < 0.05, **p < 0.01, ***p < 0.001 versus indicated group.

KAT5 is a histone acetyltransferase that is responsible for the transcriptional regulation of its target genes. AnimalTFDB analysis (http://bioinfo.life.hust.edu.cn/AnimalTFDB/#!/) predicted the potential binding sites (BS) of KAT5 to *STUB1* promoter (Fig. 6a). ChIP assay suggested that KAT5 directly bound to *STUB1* promoter (for human, BS1 and BS2; for mouse, BS2) (Fig. 6b). Furthermore, the luciferase activity of *STUB1*-WT group was strikingly enhanced after KAT5 overexpression, whereas the luciferase activity of *STUB1*-MUT group was not affected (Fig. 6c). Besides, KAT5 silencing reduced the enrichment of KAT5 and H3K27ac to the promoter of *STUB1* (Fig. 6d), indicating that KAT5 knockdown weakened the binding of H3K27ac to *STUB1*. ChIP-re-ChIP assay further proved that KAT5 directly interacted with H3K27ac on the *STUB1* promoter, which was weakened by OGD/R stimulation (Fig. 6e). To further examine the role of KAT5/STUB1 axis in MIRI, cardiomyocytes were transfected with sh-KAT5, STUB1 overexpression lentivirus, or a mixture of them. Functionally, KAT5 silencing-mediated decreased viability and increased pyroptosis of OGD/R-treated cells could be abolished by STUB1 overexpression (Fig. 6f and Supplementary Fig. 2a). Additionally, sh-KAT5-induced upregulation of Caspase-1, LATS2, p-YAP, NLRP3, ASC, IL-18, IL-1β, and downregulation of STUB1, YAP, β-catenin upon OGD/R stimulation were reversed by co-transfection with STUB1 overexpression lentivirus (Supplementary Fig. 2b–d). These observations suggested that KAT5 transcriptionally activated STUB1 to relieve OGD/R-induced cardiomyocyte injury via modulation of LATS2/YAP/β-catenin axis.

### KAT5 mitigates MIRI in mice through regulation of STUB1/LATS2/YAP/β-catenin pathway

**Fig. 7:**
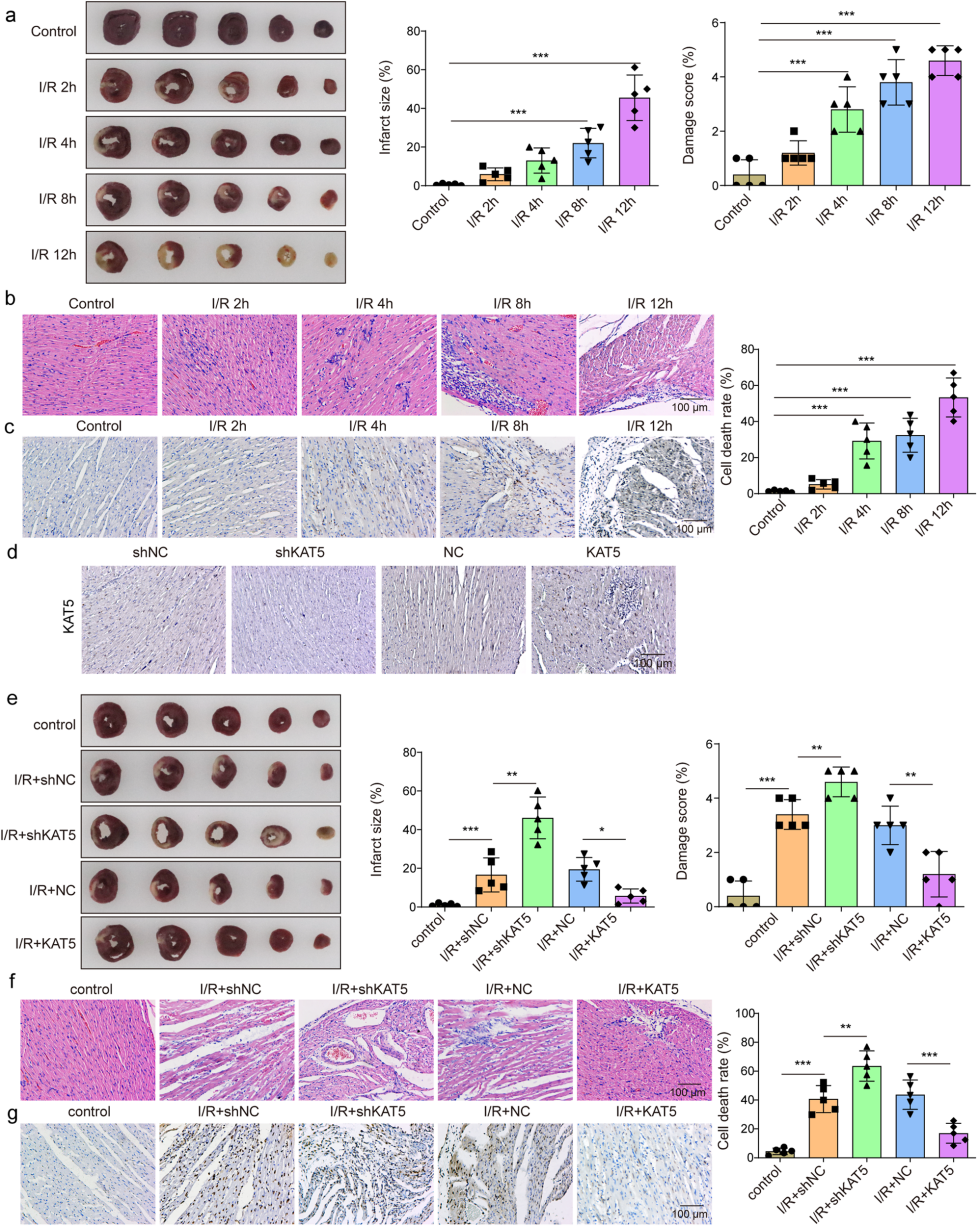
KAT5 mitigates MIRI in mice. **a** The infarct size was evaluated by TTC staining. White color indicates infarction zone, and red color indicates non-infarction zone. **b** Representative images of HE staining showed pathological changes in hearts (scale bar = 100 μm). **c** TUNEL staining evaluated the cell death in heart tissues (brown color indicates positive staining, scale bar = 100 μm). The mice were administered adenoviruses containing sh-NC, shKAT5, NC, or KAT5. **d** Immunohistochemical staining evaluated KAT5 expression in the heart tissues (brown color indicates positive staining, scale bar = 100 μm). The mice were administered adenoviruses containing sh-NC, shKAT5, NC, or KAT5 and then suffered I/R. **e** The infarct size was determined by TTC staining. White color indicates infarction zone, and the red color indicates non-infarction zone. **f** The pathological changes in hearts were observed by HE staining (scale bar = 100 μm). **g** TUNEL staining evaluated the cell death in hearts (scale bar = 100 μm). Data represent the mean ± SD. *n* = 5 animals. One-way ANOVA followed by Bonferroni was performed for statistical analysis in **a**–**j**. Box plots represent median with minimum and maximum whiskers. *p < 0.05, **p < 0.01, ***p < 0.001 versus indicated group.

**Fig. 8:**
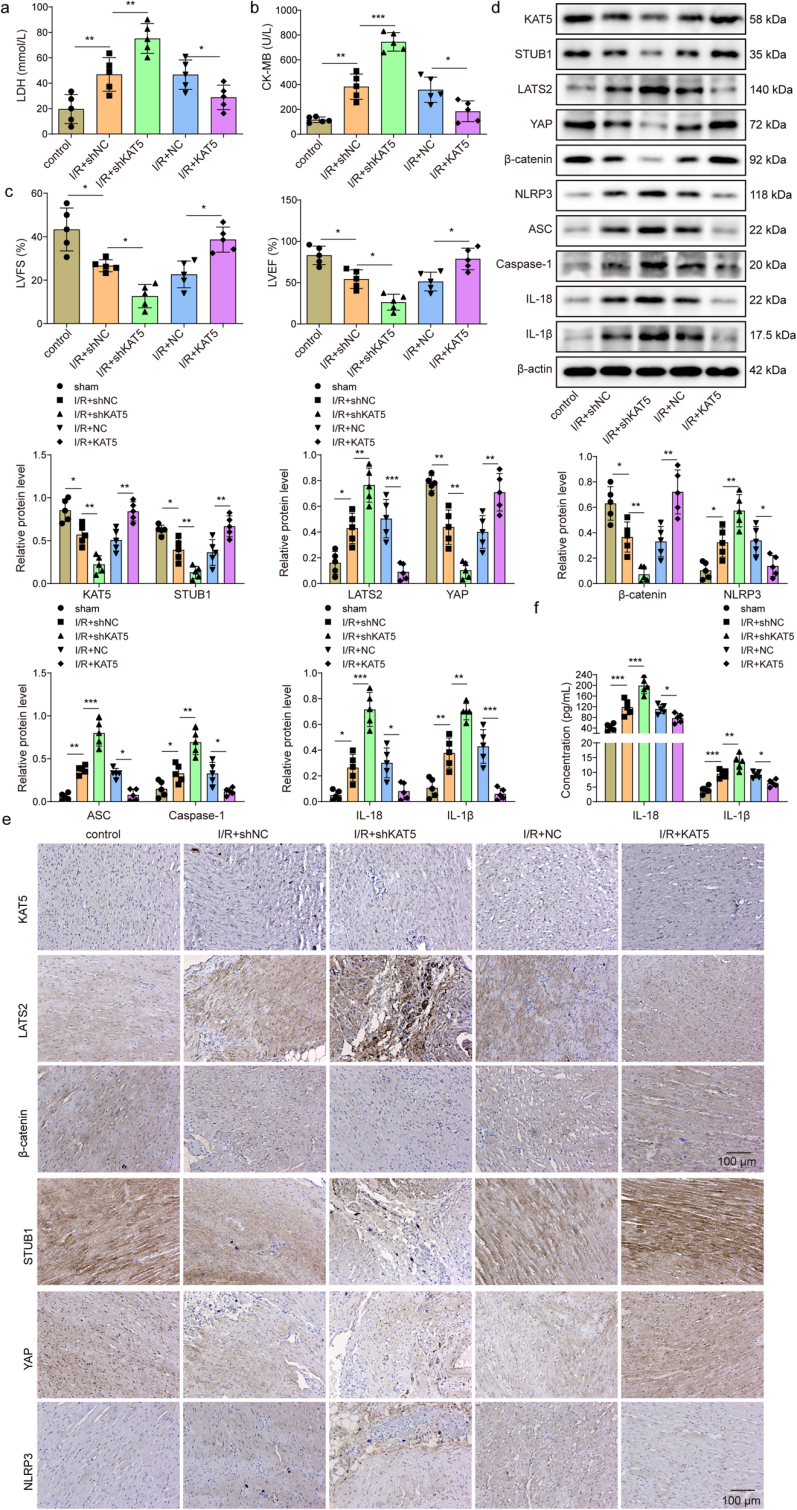
KAT5 mitigates MIRI in mice through regulation of STUB1/LATS2/YAP/β-catenin pathway. The mice were administered adenoviruses containing sh-NC, shKAT5, NC, or KAT5 and then suffered I/R. The serum activities of LDH (**a**) and CK-MB (**b**) were measured. **c** LVEF and LVFS were assessed by echocardiography. **d** Western blotting analysis of KAT5, STUB1, LATS2, YAP, β-catenin, NLRP3, ASC, Caspase-1, IL-18, IL-1β expression in heart tissues. **e** Immunohistochemical staining detected KAT5, STUB1, LATS2, YAP, β-catenin, and NLRP3 expression in the heart tissues (brown color indicates positive staining, scale bar = 100 μm). **f** The levels of IL-18 and IL-1β were measured by ELISA in heart homogenate. Data represent the mean ± SD. *n* = 5 animals. One-way ANOVA followed by Bonferroni was performed for statistical analysis in a-**c**. Box plots represent median with minimum and maximum whiskers. *p < 0.05, **p < 0.01, ***p < 0.001 versus indicated group.

Finally, we validated the in vitro results in the in vivo MIRI model. As presented in Fig. 7a, the heart infarct volume was evidently raised with the extension of reperfusion time. Besides, I/R led to pathological changes and cell death in the heart tissues of mice (Fig. 7b, c). According to the results, reperfusion for 4 h was selected in the subsequent experiments. Adenoviruses-mediated knockdown of KAT5 and overexpression of KAT5 in the heart tissues were confirmed by immunohistochemical staining (Fig. 7d). Administration with adenoviruses containing sh-KAT5 remarkably enhanced infarct volume, aggravated pathological changes, and apoptosis; however, we obtained contrary results after treatment with containing KAT5 overexpression adenoviruses (Fig. 7e–g). Moreover, I/R-induced increased LDH and CK-MB activities, and decreased LVEF and LVFS were intensified by KAT5 silencing, but suppressed by KAT5 overexpression (Fig. 8a–c). In consistent with the in vitro data, KAT5 silencing further promoted I/R-induced increased expression of LATS2, NLRP3, ASC, Caspase-1, IL-18, IL-1β, and reduced expression of KAT5, STUB1, YAP, β-catenin in the hearts, whereas KAT5 overexpression resulted in the opposite results (Fig. 8d–f). Collectively, KAT5 mitigated MIRI in vivo via regulating STUB1/LATS2/YAP/β-catenin axis.

## Discussion

MIRI is one of the most common cardiovascular diseases and as well as the main reason for death worldwide. Genetic investigations have suggested the contribution of Hippo-YAP pathway activation to myocardium repair and regeneration after MIRI^20^. Overexpression of YAP in cardiomyocytes facilitated cardiac regeneration after MIRI partly through the activation of AKT pathway^21^. The modulation of upstream and downstream molecules of YAP pathway might influence the progression of MIRI. For instance, depletion of LATS2, an upstream regulator of the YAP pathway, protected against MIRI by enhancing cardiomyocyte viability^22^. It has been reported that promotion of LATS2 expression facilitated the phosphorylation of YAP, and then restrained YAP nuclear translocation, thereby inhibiting YAP-mediated transcriptional regulation of genes^23^. In another study, activation of β-catenin by YAP repressed cardiomyocyte hypertrophy after MIRI^24^. In line with these previous studies, we demonstrated that LATS2 knockdown alleviated MIRI by suppressing NLRP3-mediated pyroptosis through activating YAP/β-catenin signaling.

As an E3 ubiquitin ligase, STUB1 is upregulated in heart tissues, which exerts key roles in protein quality control^13^. STUB1 has been identified to attenuate toxicity via proteasome-dependent degradation of target protein^25^. Tang et al. suggested that STUB1 activation prevented necroptosis during cerebral ischemia-reperfusion injury through degradation of RIPK1 and RIPK3 proteins^26^. Notably, STUB1 was demonstrated as a potent cardioprotective protein in high glucose-induced cardiac injury^27^. STUB1 also mitigated MIRI via promoting p53 degradation^28^. In this work, we demonstrated that STUB1 was downregulated in MIRI models. We also revealed that STUB1 directly interacted with LATS2 to promote proteasome-dependent degradation of LATS2 protein, which repressed NLRP3-mediated pyroptosis of cardiomyocytes and ameliorated MIRI via activation of YAP/β-catenin pathway.

KAT5 is a lysine acetyltransferase that is known to regulate the acetylation of its target proteins. Studies have reported that KAT5 takes part in the regulation of various biological processes, including cell cycle, DNA injury, and inflammatory response^29,30^. The involvement of KAT5 in multiple disorders has also been documented. For example, KAT5 was lowly expressed in rheumatoid arthritis T cells, which affected regulatory T cell function via reducing Foxp3 acetylation level^31^. Song et al. suggested that KAT5 promoted the acetylation of DNA sensor cGMP-AMP synthase, which enhanced initiating innate immune response to DNA virus^32^. A recent study demonstrated that KAT5 meliorated ischemic acute kidney injury through epigenetic modulation of KCC3 expression^11^. Our study validated that KAT5 recruited H3K27ac to bind to STUB1 promoter and epigenetically increased STUB1 expression. Further findings suggested that KAT5 delayed MIRI development through the regulation of STUB1/LATS2/YAP/β-catenin signaling. Our results provided first evidence for the protective mechanisms of KAT5 in MIRI.

The major limitation of this study is that the quality for immunohistochemical staining is not ideal. The staining quality can be affected by various factor, such as the heating-up period for antigen retrieval, antibody dilution, antibody incubation time, DAB staining time, and so on. In addition, to avoid false positive result, the positive and negative control sections can be used. In our future study, we will further optimize the experimental conditions to improve the result quality.

Taken together, we demonstrated that KAT5 raised STUB11 expression through acetylation and subsequently modulated LATS2-YAP-β-catenin axis to relieve MIRI. KAT5 might be a potential therapeutic target for MIRI.

## Materials and methods

### Cell culture and transfection

Mouse atrial cardiomyocytes (HL-1, Cat. No.: SCC065) were obtained from Sigma-Aldrich (USA). Human cardiomyocytes (AC16, Cat. No.: CRL-3568) were provided by the American Type Culture Collection (USA). HL-1 and AC16 cells were authenticated by STR profiling and tested for mycoplasma contamination. All cells were cultured in DMEM (Gibco, USA) containing 10% fetal bovine serum (Gibco) with 5% CO_2_ at 37 °C.

ShRNAs targeting human *LATS2* (sh-*LATS2*, sequences: 5′-cgtccattacattgacttcaa-3′), *KAT5* (sh-*KAT5*, sequences: 5′-gccatgaagaccctaaggaaa-3′) and *STUB1*(*sh-STUB1*, sequence: 5′-ttggctatgaaggaggttatt-3′), mouse *Lats2* (sh-*Lats2*, sequences: 5′-ccgaagtttggaccttatcaa-3′), *Kat5* (sh-*Kat5*, sequences: 5′-cctcctatcctaccgaagtta-3′) and *Stub1*, (sh-*Stub1*, sequences: 5′-cacgataaatacatggcagat-3′) (see supplementary table 1 for details), overexpression lentivirus for human LATS2, STUB1 genes and mouse *Lats2* and *Kat5* genes were obtained from GenePharma (Shanghai, China). Besides, overexpression plasmids for human GCN5 and CBP genes were obtained from GenePharma (Shanghai, China). HL-1 and AC16 cells were transfected with these segments using Lipofectamine2000 (Thermo Fisher, USA).

### In vitro MIRI model

The in vitro MIRI model was established by oxygen-glucose deprivation/reoxygenation (OGD/R) as previously described^33^. In brief, AC16 and HL-1 cells were suspended in glucose- and serum-free DMEM, followed by culture in a hypoxic chamber (1% O_2_, 94% N_2_, and 5% CO_2_) at 37 °C for 2, 4, 8, or 12 h. Afterward, the cells were maintained in DMEM containing 10% FBS under normal conditions (95% air and 5% CO_2_) for 6 h for reoxygenation.

### Cell counting Kit-8 (CCK-8)

The viability of AC16 and HL-1 cells was measured using CCK-8 reagent (Cat. No.: CK04, Dojindo, Japan). After various treatments, the cells were planted into 96-well plates at a density of 10,000 cells per well. Thereafter, the cells were added with 10 μL of CCK-8 reagent and reacted for 1 h at 37 °C. The absorbance at 450 nm was detected on a microplate reader (Thermo Fisher) to evaluate cell viability.

### Animal model

Six- to eight-week-old male C57BL/6 mice were provided by SLAC Laboratory Animal Co., Ltd (Shanghai, China). To establish the in vivo MIRI model, the mice were anesthetized with 2% isoflurane, and then the chest of mice was opened to expose the hearts. The left anterior descending coronary artery was ligated with a 9‐0 ophthalmic suture. The pale color in the myocardium indicated a successful occlusion. After ligation for 45 min, the blood flow was restored for 2, 4, 8, and 12 h, respectively. The same surgical procedure without silk ligation was performed on the sham mice. The I/R mice were randomly divided into four groups: sh-NC, sh-KAT5, NC, KAT5. At one week before I/R surgery, thoracotomy was performed for the injection of 10 µL adenoviruses (1 × 10^10^ VP/mL, GenePharma) containing sh-NC, sh-KAT5, NC, or KAT5 to the apex of the hearts using a 30 G injection needle^34^. There were 5 mice in each experimental group. At 2, 4, 8, and 12 h after reperfusion, the mice were euthanized by cervical dislocation and the heart tissue samples were collected. All animal experiments were approved by the Ethics Committee of Anhui Medical University (LLSC20221093). We have complied with all relevant ethical regulations for animal use.

### Echocardiography

To evaluate heart function of mice, echocardiography by VEVO2100 ultrasound imaging system (VisualSonics, Canada) was performed after anesthetization with 2% isoflurane. The probe was placed on the left chest after removal of chest hair and then echocardiographic images were detected in M mode. Consequently, the left ventricular ejection fraction (LVEF) and fractional shortening (LVFS) were assessed.

### 2, 3, 5-triphenyltetrazolium chloride (TTC) staining

Myocardial infarct size was measured by TTC staining. The hearts were excised, perfused with 1% Evans blue and cut into 5 slices, followed by staining with 1% TTC for 15 min. The images were taken and the infarct area was quantified by ImageJ software.

### Hematoxylin-eosin (HE) staining

The heart tissues were fixed with 4% paraformaldehyde overnight. After paraffin-embedding, the heart tissues were cut into 5 μm slices. Then, the slices were stained with hematoxylin for 3 min and then stained with eosin for 3 min. A light microscope (Olympus) was adopted to take micrographs.

### Immunohistochemical staining

Heart tissue sections were deparaffinized and rehydrated for 20 min with xylene and graded ethanol solution. Antigen retrieval was performed using boiling 0.01 M citrate buffer. Endogenous peroxidase activity was suppressed by incubation with 3% hydrogen peroxide. All sections were incubated with primary antibodies against KAT5 (bs-13686R, 1:100, Bioss, Beijing, China), STUB1 (ab134064, 1:100, Abcam, UK), LATS2 (bs-4081R, 1:100, Bioss), YAP (bs-3605R, 1:100, Bioss), β-catenin (A19657, 1:50, Abclonal), and NLRP3 (MA5-32255, 1:50, Thermo Fisher) overnight at 4 °C. After thorough washing, the sections were incubated with biotin-conjugated secondary antibody for 30 min and colored with di-amino benzidine. All sections were counterstained with hematoxylin and imaged using a light microscope.

### Detection of lactate dehydrogenase (LDH) and creatine kinase (CK-MB)

The serum levels of LDH and CK-MB were assessed using the Lactate dehydrogenase assay kit (Cat. No.: A020-2-2) and creatine kinase assay kit (Cat. No.: H197-1-2) purchased from Nanjing Jiancheng Bioengineering Institute according to the instructions, respectively.

### TdT-mediated dUTP nick end labeling (TUNEL)

To assess cardiomyocyte apoptosis, TUNEL was performed. In brief, the heart tissue sections were fixed in 4% paraformaldehyde, followed by staining with TUNEL Assay Kit- HRP-DAB (Cat. No.: ab206386, Abcam, UK) for heart sections following the manufacturer’s protocols. TUNEL-positive cell percentage was quantified using ImageJ software.

### Flow cytometry

Pyroptosis of cells was detected by flow cytometry using propidium iodide (PI) and Caspase-1 staining. Briefly, each group of cells were collected after centrifugation at 1000 rpm for 5 min. Then, cells were stained with the Caspase-1 probe at 37 °C for 1 h, followed by staining with PI for 10 min. The stained cells were detected on a flow cytometer (Thermo Fisher). Pyroptotic cells were gated as Caspase-1+/PI+ (Supplementary Fig. 3). The pyroptosis percentage was calculated as the percentage of double-positive staining of Caspase-1 and PI.

### Quantitative real-time polymerase chain reaction (RT-qPCR)

TRIzol reagent (Thermo Fisher) was utilized for the isolation of total RNA, followed by cDNA synthesis using the RT MasterMix for qPCR II (Cat. No.: HY-K0510A, MCE). RT-qPCR was carried out using specific primers and the 2×SYBR Green PCR Mastermix (Cat. No.: SR1110, Solarbio, Beijing, China). Gene levels were analyzed using the 2^−ΔΔCT^ method with *GAPDH* as the internal control. Primer sequences are shown in supplementary table 2.

### Western blotting

RIPA buffer (Solarbio) was used for total protein extraction, and the protein concentration was assessed by BCA Protein Assay Kit (Cat. No.: PC0020, Solarbio). The protein samples were separated by sodium dodecyl sulfate polyacrylamide gel electrophoresis, and transferred to polyvinylidene fluoride membranes, followed by blocking in 5% skim milk for 1 h. Subsequently, the membranes were probed with primary antibodies against KAT5 (bs-13686R, 1:500, Bioss), STUB1 (A11751, 1:1000, Abclonal, Wuhan, China), LATS2 (bs-4081R, 1:500, Bioss), p-YAP (bsm-52214R, 1:500, Bioss), YAP (bs-3605R, 1:1000, Bioss), β-catenin (A19657, 1:1000, Abclonal), NLRP3 (A5652, 1:500, Abclonal), ASC (bs-6741R, 1:500, Bioss), Caspase-1 (A0964, 1:1000, Abclonal), IL-18 (A1115, 1:500, Abclonal), IL-1β (A16288, 1:500, Abclonal), β-actin (bs-0061R, 1:5000, Bioss) overnight at 4 °C. After incubation with the secondary antibody for 1 h, the protein bands were developed by the ECL Western Blotting Substrate (Cat. No.: PE0010, Solarbio). For tissue detection, five samples from each group were used. For cell detection, three samples from three independent experiments were used.

### Immunofluorescence staining

AC16 and HL-1 cells were planted on coverslips. After fixing with 4% paraformaldehyde, the cells were penetrated with 0.3% Triton X-100, blocked in 3% BSA, and probed with primary antibody against caspase-1 (A0964, 1:50, Abclonal) at 4 °C overnight. Then, the cells were dropped with Cy3 Goat Anti-Rabbit IgG (1:100, Abclonal) and incubated for 1 h. After staining with DAPI, the fluorescence was observed under a fluorescence microscope. Caspase-1 positive cell percentage was quantified with ImageJ software.

### Enzyme-linked immunosorbent assay (ELISA)

IL-18 and IL-1β release from AC16 and HL-1 cells was assessed using commercial Human IL-18 ELISA kit (Cat. No.: SEKH-0028), Mouse IL-18 ELISA kit (Cat. No.: SEKM-0019), Human IL-1β ELISA kit (Cat. No.: SEKH-0002), and Mouse IL-1β ELISA kit (Cat. No.: SEKM-0002) purchased from Solarbio.

### Co-immunoprecipitation (Co-IP)

To evaluate the interaction between exogenous STUB1 and LATS2 proteins, Myc-LATS2 and SFB-Flag-STUB1 plasmids were co-transfected into HEK293T cells using Lipofectamine2000. After the transfection for 24 h, HEK293T cells were lysed with IP lysis buffer. Then, cell lysates were immunoprecipitated using anti-SFB agarose beads. After washing with lysis buffer, LATS2 and Flag protein levels were assessed by Western blotting.

For the detection of interaction between endogenous STUB1 and LATS2 proteins or ubiquitination of LATS2, AC16, and HL-1 cells were lysed with the IP lysis buffer. The cell lysates were pre‐cleaned with protein A/G beads at 4 °C for 4 h, and immunoprecipitated with the protein A/G beads conjugated with anti-STUB1 (A11751, 4 μg, Abclonal), anti-Ubiquitin (sc-8017, 2 μg, Santa Cruz, USA), or anti-IgG (AC005, 4 μg, Abclonal) antibody at 4 °C overnight. Finally, the bound protein to A/G beads was washed with IP lysis buffer and detected by Western blotting.

### Chromatin immunoprecipitation (ChIP) and re-ChIP

ChIP assay was performed using the ChIP assay kit (Cat. No.: 17-371, Millipore, USA). In brief, cells were treated with 1% formaldehyde for 10 min and then incubated with 125 mM glycine for 5 min. Cell lysates were prepared using cell lysis buffer and then subjected to sonication. Thereafter, immunoprecipitation was performed using anti-KAT5 (NBP2-20647, 1:50, Novus Biologicals, USA), anti-H3K27Ac (A-4708-050, 1:50, Epigentek, USA), or anti-IgG (AC005, 4 μg, Abclonal) overnight at 4  °C, and then the precipitates were incubated with protein G magnetic beads for 2 h at 4  °C. The protein-DNA complexes were eluted and the purified DNAs were measured by qPCR. For Re-ChIP, the eluted protein-DNA complexes by the first immunoprecipitation using anti-H3K27Ac were re-immunoprecipitated with anti-KAT5 or anti-IgG overnight at 4  °C, followed by detection by qPCR. Primer sequences of *STUB1* promoter are shown in supplementary table 3.

### Dual-luciferase reporter assay

Wild-type (WT) or mutated (MUT) *STUB1* promoter sequences were inserted into the pGL3-luciferase reporter vector. AC16 and HL-1 cells were transfected with the established luciferase reporter plasmids together with sh-NC or sh-KAT5. After transfection for 48 h, the luciferase activity was determined using a Dual-Luciferase Reporter Gene Assay Kit (Cat. No.: RG027, Beyotime, Haimen, China).

### Statistics and reproducibility

All experiments were performed in at least three biological replicates, and each biological replicate contained three technical replicates. All values are presented as mean ± standard deviation (SD) and analyzed by GraphPad Prism 8.0 software. The normality distribution of data was evaluated using the Shapiro–Wilk’s test. Detailed sample sizes are described in the figure legends. Two-tailed unpaired Student’s *t* test was performed to evaluate the differences between two groups of RT-qPCR, Western blotting, dual-luciferase reporter assay, and ChIP data. One-way analysis of variance (ANOVA) followed by Bonferroni post-hoc test was performed to evaluate differences among more than 2 groups of CCK-8, flow cytometry, Western blotting, RT-qPCR, ELISA, TTC staining, HE staining, TUNEL staining, immunohistochemical staining, serum biochemical indices, and echocardiography data. *P* < 0.05 was considered as statistically significant.

### Reporting summary

Further information on research design is available in the Nature Portfolio Reporting Summary linked to this article.

### Supplementary information

Supplementary Information

Description of Additional Supplementary Files

Supplementary Data

Reporting Summary

## Data Availability

Source data for the graphs are available as a Supplementary Data file. The sequences of shRNAs, primers used in RT-qPCR and ChIP methods, and all of the uncropped images in western blotting were shown in the Supplementary information document. To be specific, that the mRNA level of LATS2 was not affected by OGD/R stimulation or STUB1 overexpression was shown in Supplementary Fig. 1, and that KAT5 acetylates STUB1 to relieve OGD/R-induced cardiomyocyte injury were presented in Supplementary Fig. 2, the schematic drawing of pyroptotic cells gated as Caspase-1+/PI+ was showed in Supplementary Fig. 3. Besides, uncropped and unedited Western blot/gel images were included in Supplementary Fig. 4. The sequences of shRNAs were shown in Supplementary Table 1. Primers used in RT-qPCR methods are shown in Supplementary Table 2. Primers used in ChIP methods are shown in Supplementary Table 3. The other data generated and/or analyzed during the current study are available from the corresponding author upon reasonable request.
