# Supplementary material for: STUB1 is acetylated by KAT5 and alleviates myocardial ischemia-reperfusion injury through LATS2-YAP-β-catenin axis
Source: Commun Biol. 2024 Apr 1;7:396. doi: 10.1038/s42003-024-06086-9 (PMC10985082; doi:10.1038/s42003-024-06086-9)
Supplement: Supplementary file 1 — Supplementary Information [file 42003_2024_6086_MOESM1_ESM.pdf]

Supplementary Informmation

1. Supplementary Figure and Figure legend

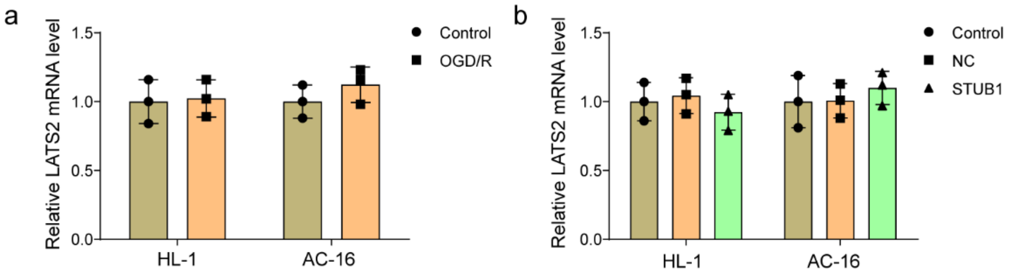

Supplementary Figure 1. The mRNA level of LATS2 was not affected by OGD/R stimulation or STUB1 overexpression. (a) *LATS2* mRNA level in OGD/R-stimulated AC16 and HL-1 cells was detected by RT-qPCR. (b) AC16 and HL-1 cells were transfected with NC or STUB1 overexpression plasmid, and *LATS2* expression was assessed by RT-qPCR. Data represent the mean ± SD. n=3 independent experiments. Student's t test (for A) and one-way ANOVA followed by Bonferroni (for B) were performed for statistical analysis. Box plots represent median with minimum and maximum whiskers.

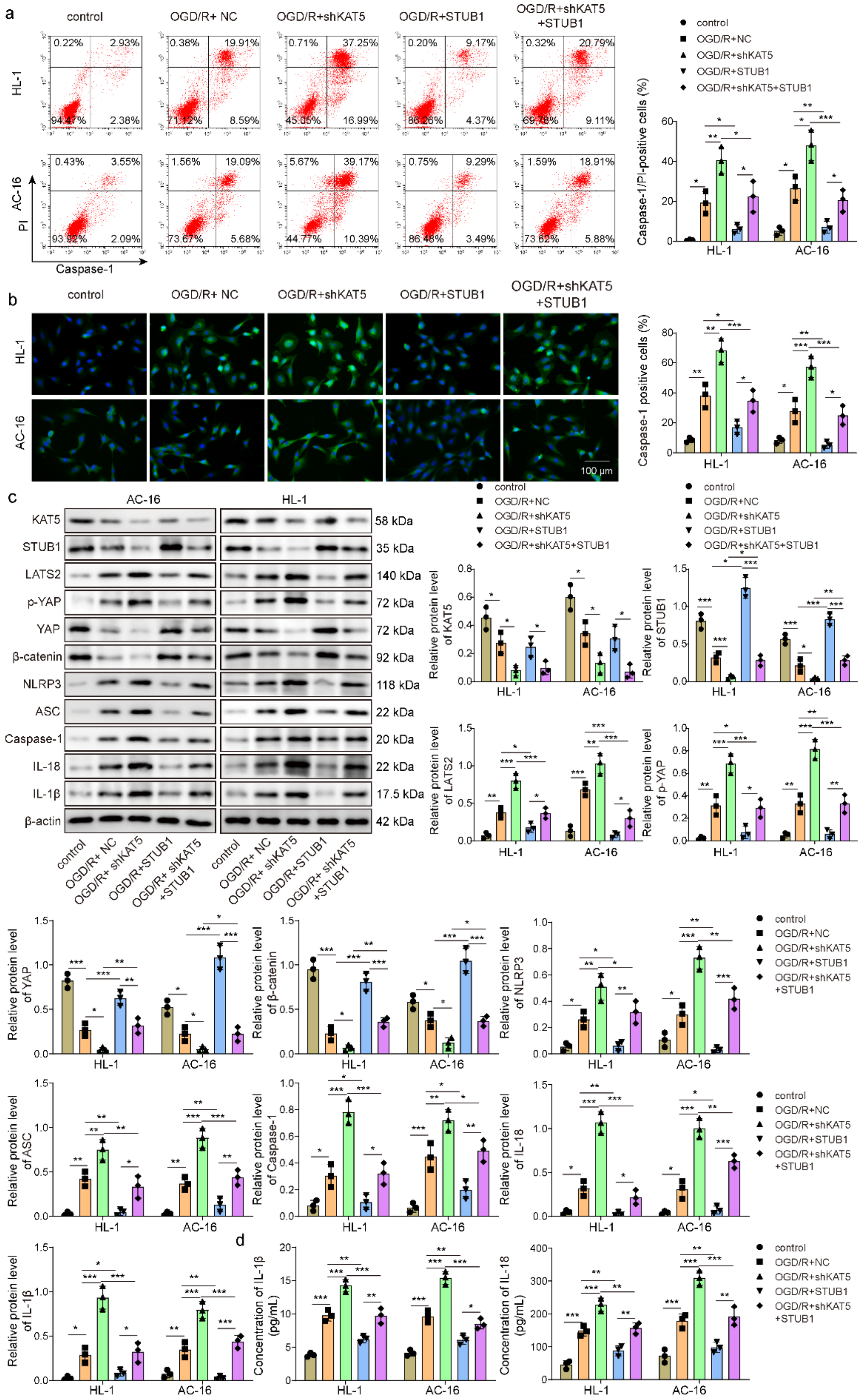

Supplementary Figure 2. KAT5 acetylates STUB1 to relieve OGD/R-induced cardiomyocyte injury. (a) Flow cytometry was adopted to measure cell pyroptosis. (b) Caspase-1 expression was detected by immunofluorescence staining (green

fluorescence). Scale bar = 100 μm. (c) KAT5, STUB1, LATS2, p-YAP, YAP, β-catenin, NLRP3, ASC, Caspase-1, IL-18, IL-1β levels were assessed by Western blotting. (d) IL-18 and IL-1β production was measured by ELISA. Data represent the mean ± SD. Each experiment should be repeated independently at least three times. n=3 independent experiments. For a-d, one-way ANOVA followed by Bonferroni was performed for statistical analysis. Box plots represent median with minimum and maximum whiskers. $*p < 0.05$, $**p < 0.01$, $***p < 0.001$ versus indicated group.

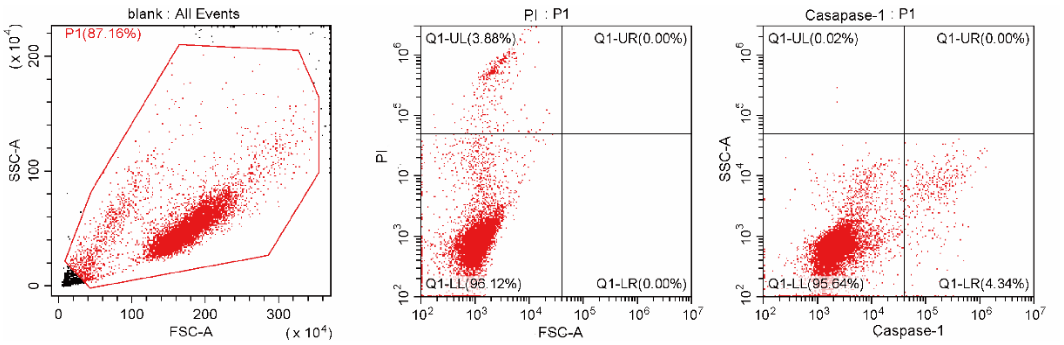

Supplementary Figure 3. The schematic drawing of pyroptotic cells gated as Caspase-1+/PI+ in flow cytometry method.

1c

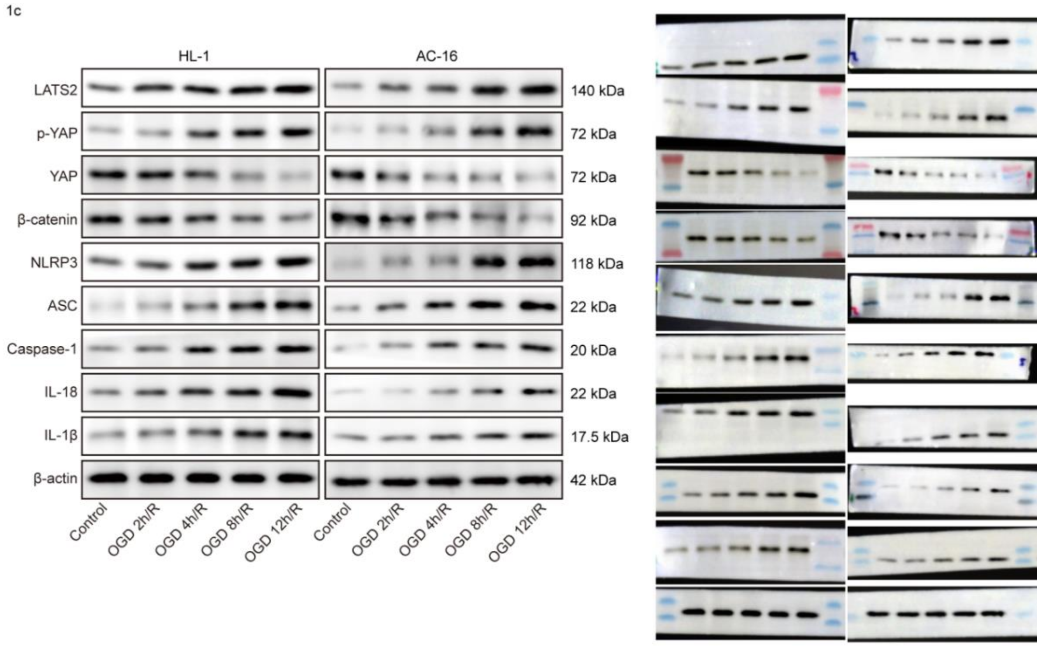

HL-1
AC-16
LATS2
p-YAP
YAP
β-catenin
NLRP3
ASC
Caspase-1
IL-18
IL-1β
β-actin
140 kDa
72 kDa
72 kDa
92 kDa
118 kDa
22 kDa
20 kDa
22 kDa
17.5 kDa
42 kDa
Control
OGD 2h/R
OGD 4h/R
OGD 8h/R
OGD 12h/R
Control
OGD 2h/R
OGD 4h/R
OGD 8h/R
OGD 12h/R

2b

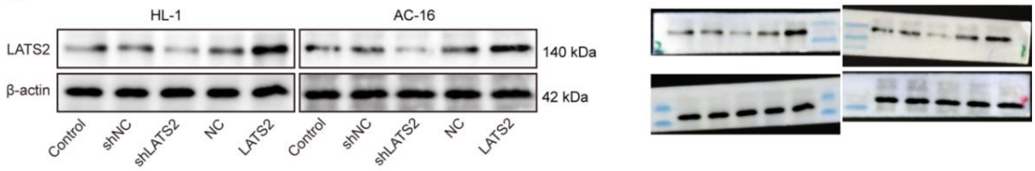

HL-1
AC-16
LATS2
β-actin
140 kDa
42 kDa
Control
shNC
shLATS2
NC
LATS2
Control
shNC
shLATS2
NC
LATS2

2f

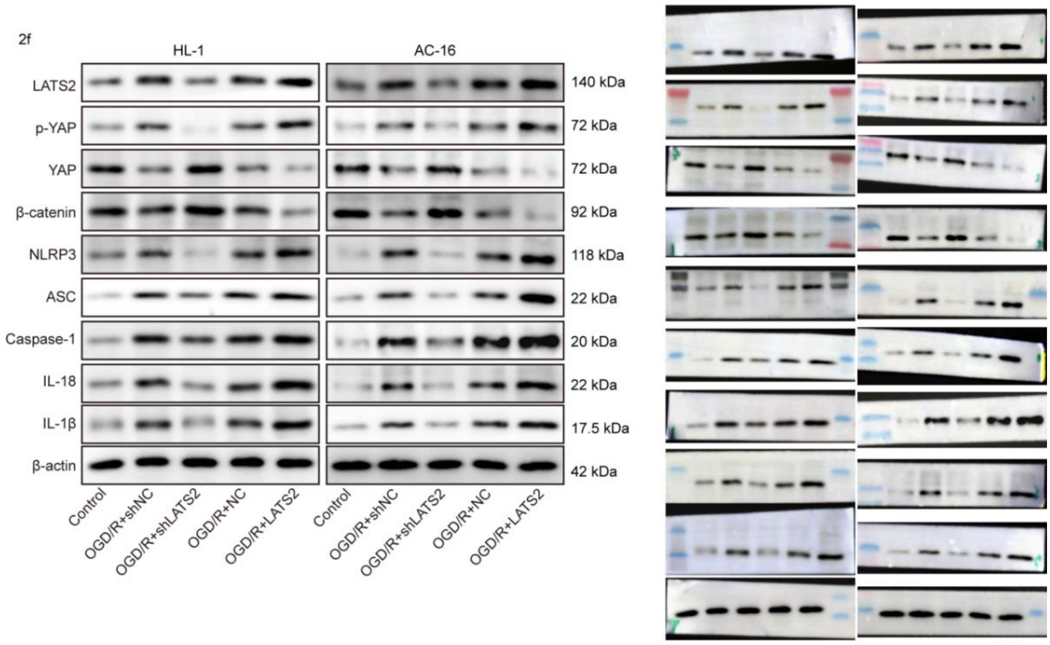

HL-1
AC-16
LATS2
p-YAP
YAP
β-catenin
NLRP3
ASC
Caspase-1
IL-18
IL-1β
β-actin
140 kDa
72 kDa
72 kDa
92 kDa
118 kDa
22 kDa
20 kDa
22 kDa
17.5 kDa
42 kDa
Control
OGD/R+shNC
OGD/R+shLATS2
OGD/R+NC
OGD/R+LATS2
Control
OGD/R+shNC
OGD/R+shLATS2
OGD/R+NC
OGD/R+LATS2

3c

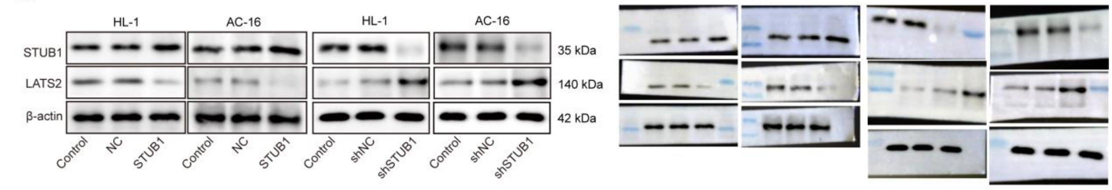

3d

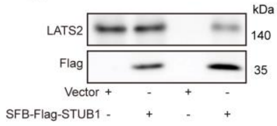

3e

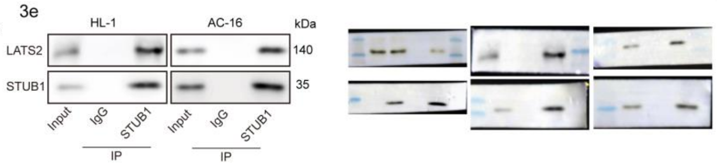

3f

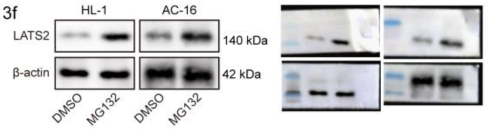

3g

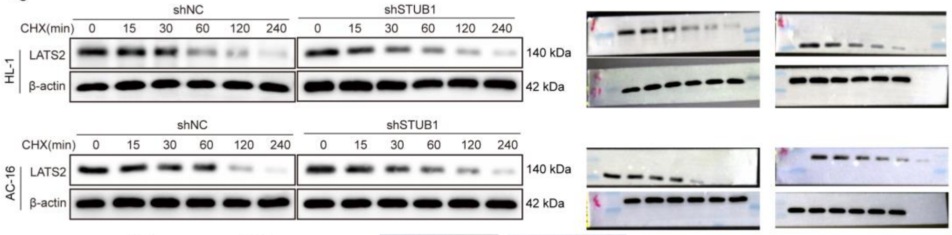

3h

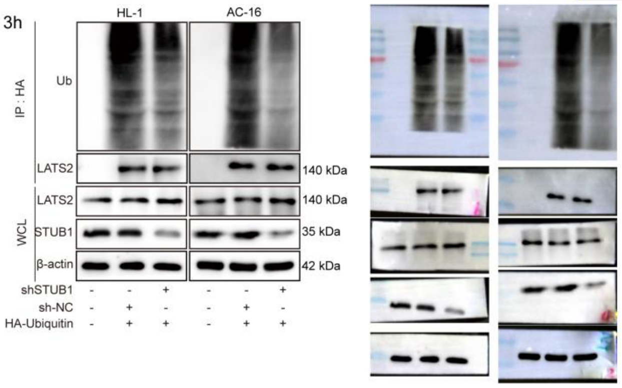

4d

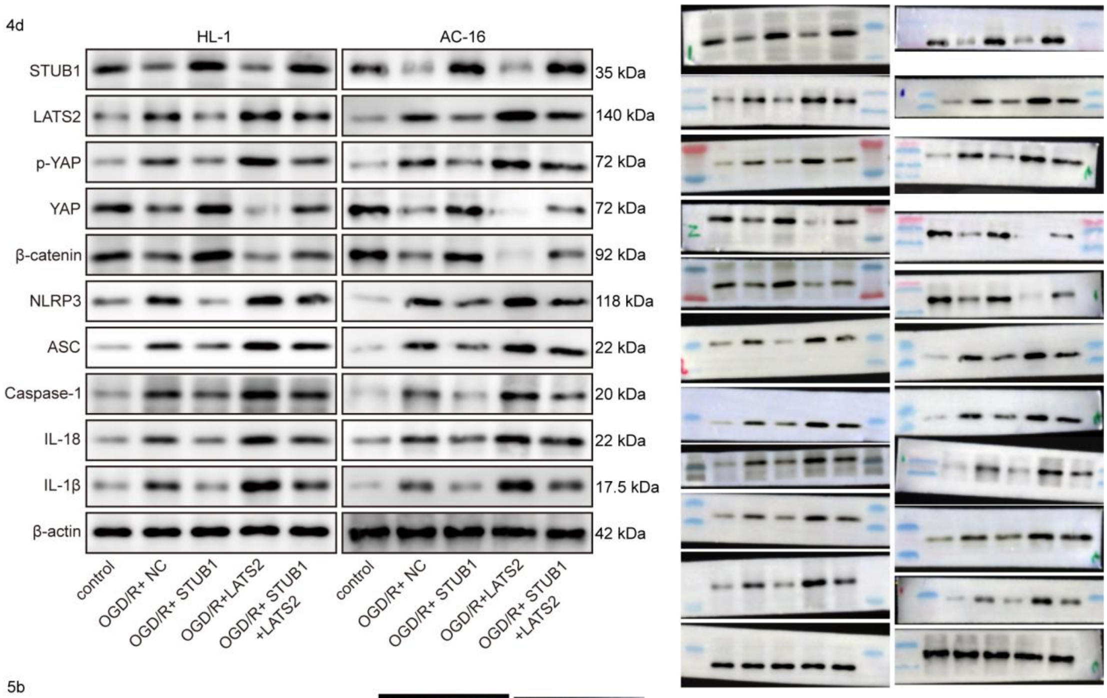
HL-1
AC-16
STUB1
LATS2
p-YAP
YAP
β-catenin
NLRP3
ASC
Caspase-1
IL-18
IL-1β
β-actin
35 kDa
140 kDa
72 kDa
72 kDa
92 kDa
118 kDa
22 kDa
20 kDa
22 kDa
17.5 kDa
42 kDa
control
OGD/R+ NC
OGD/R+ STUB1
OGD/R+LATS2
OGD/R+ STUB1 +LATS2

5b

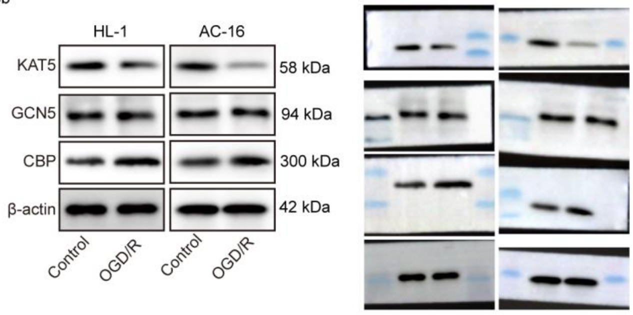
HL-1
AC-16
KAT5
GCN5
CBP
β-actin
58 kDa
94 kDa
300 kDa
42 kDa
Control
OGD/R

5e

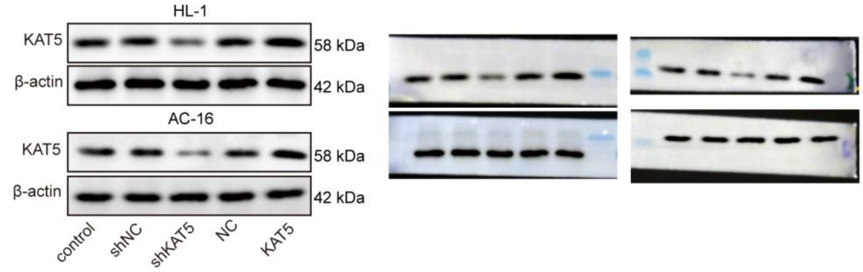
HL-1
KAT5
β-actin
58 kDa
42 kDa
AC-16
control
shNC
shKAT5
NC
KAT5

5i

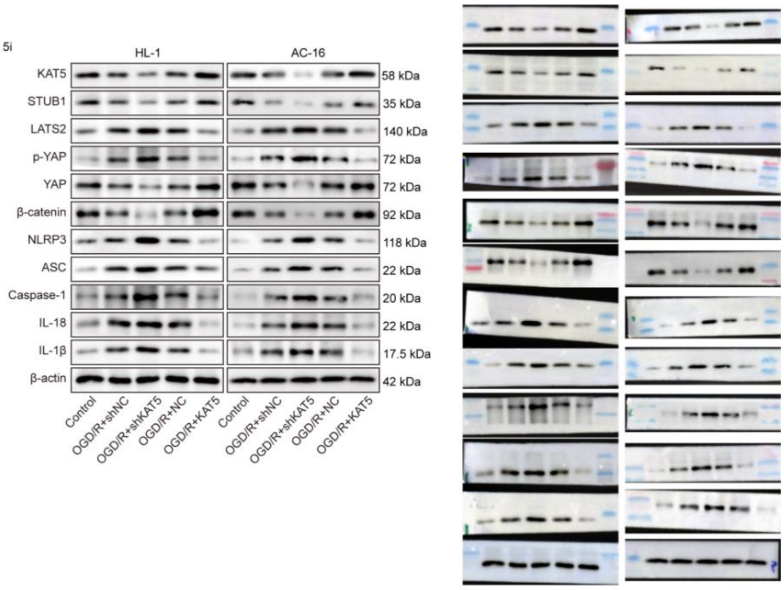

6i

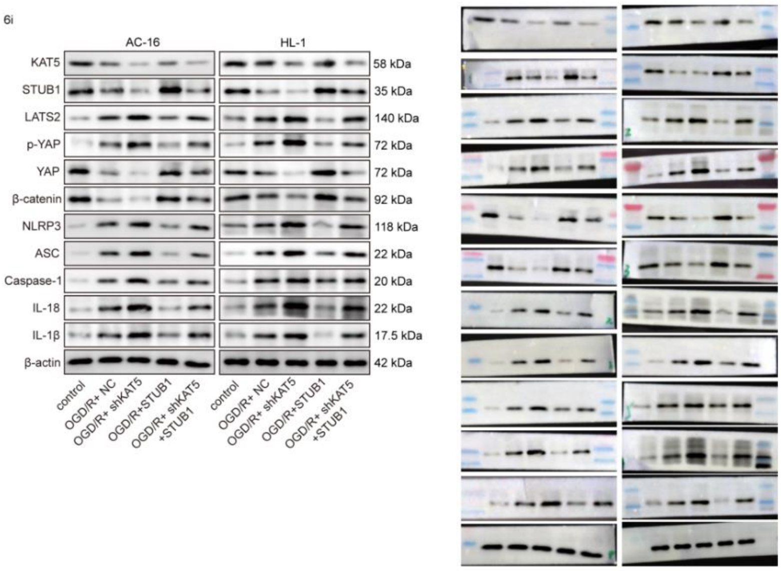

8d

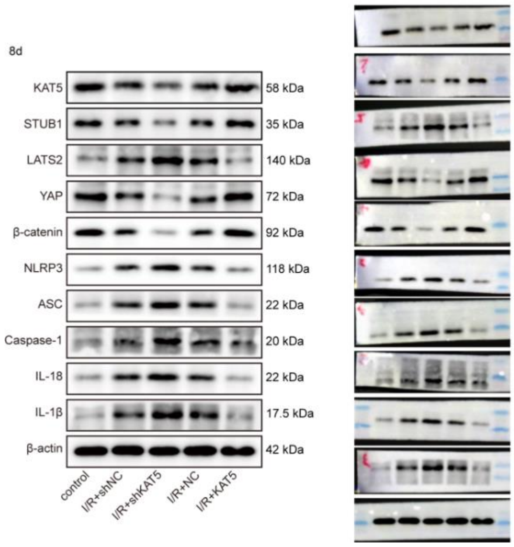

Supplementary Figure 4 Uncropped and unedited Western blot/gel images

2. Supplementary Table 1 Targets and sequences related to shRNAs used in cell transfection

Human shKAT5	cgtccattacattgacttcaa
S	AATTGcgtccattacattgacttcaaTTCAAGAGAttgaagtcaatgtaatggacgTTTTTT
A	GATCAAAAAAcgtccattacattgacttcaaTCTCTTGAAttgaagtcaatgtaatggacgC
Murine shKat5	cctcctatcctaccgaagtta
S	AATTGcctcctatcctaccgaagttaTTCAAGAGAtaacttcggtaggataggaggTTTTTT
A	GATCAAAAAAcctcctatcctaccgaagttaTCTCTTGAAtaacttcggtaggataggaggC
Human shLATS2	gccatgaagaccctaaggaaa
S	AATTGgccatgaagaccctaaggaaaTTCAAGAGAtttccttagggtcttcatggcTTTTTT
A	GATCAAAAAAgccatgaagaccctaaggaaaTCTCTTGAAtttccttagggtcttcatggcC
Murine ShLats2	ccgaagtttggaccttatcaa
S	AATTGccgaagtttggaccttatcaaTTCAAGAGAttgataaggtccaaacttcggTTTTTT
A	GATCAAAAAAccgaagtttggaccttatcaaTCTCTTGAAttgataaggtccaaacttcggC
Human shSTUB1	ttggctatgaaggaggttatt
S	AATTGttggctatgaaggaggttattTTCAAGAGAaataacctccttcatagccaaTTTTTT
A	GATCAAAAAAttggctatgaaggaggttattTCTCTTGAAaataacctccttcatagccaaC
Murine shStub1	cacgataaatacatggcagat
S	AATTGcacgataaatacatggcagatTTCAAGAGAatctgccatgtatttatcgtgTTTTTT
A	GATCAAAAAAcacgataaatacatggcagatTCTCTTGAAatctgccatgtatttatcgtgC

3. Supplementary Table 2 Oligonucleotide primer sets for qPCR

Name	Sequence (5'¬3')	Length
Human *LATS2* F	ACAAGATGGGCTTCATCCAC	20
Human *LATS2* R	CTCCATGCTGTCCTGTCTGA	20
Murine *Lats2* F	AGCAGATTGTGCGAGTCATC	20

Murine *Lats2* R	GTGGTAGGATGGGAGTGCTT	20
Human *STUB1* F	TCAAGGAGCAGGGCAATCGTCT	22
Human *STUB1* R	GCATCTTCAGGTAGCACAAGGC	22
Murine *Stub1* F	CATATCTCACCAGGCTCATTGC	22
Murine *Stub1* R	TATCTGCCATGTATTTATCGTGCTTG	26
Human *KAT5* F	CAACCACCGCTCAACGAAAC	20
Human *KAT5* R	AGAAGTACCACGGCTTGAGG	20
Murine *Kat5* F	CCACAAGAGCTTACCACGCT	20
Murine *Kat5* R	AGGGTGCCGAAGATCACATT	20
Human *GAPDH* F	AGGTCGGAGTCAACGGATTT	20
Human *GAPDH* R	TGACGGTGCCATGGAATTTG	20
Murine *Gapdh* F	AGGTCGGTGTGAACGGATTTG	21
Murine *Gapdh* R	TGTAGACCATGTAGTTGAGGTCA	23

4. Supplementary Table 3 The primers used in ChIP methods

Name	Sequence (5'¬3')	Length
Human *STUB1-BS1* F	gaactggagcgttcctcccag	21
Human *STUB1-BS1* R	cagcgtcttcatctccaccttc	22
Human *STUB1-BS2* F	cctatgtacccaagtcgtgcc	21
Human *STUB1-BS2*-R	cacgtctgcagcagctggtg	20
Human *STUB1-BS3* F	ctgggccacaggtgctg	17
Human *STUB1-BS3* R	ctcaggcctccgggtctcac	20
Murine *Stub1-BS1* F	gaaaccacgcagcgaagtttg	21
Murine *Stub1-BS1* R	ggctggtttccttggagagcg	21
Murine *Stub1-BS2* F	ctgaagccaagtcttactgcagc	23
Murine *Stub1-BS2* R	gacagctacctggtgctgtg	20
