# Supplementary material for: STUB1 is acetylated by KAT5 and alleviates myocardial ischemia-reperfusion injury through LATS2-YAP-β-catenin axis
Source: Commun Biol. 2024 Apr 1;7:396. doi: 10.1038/s42003-024-06086-9 (PMC10985082; doi:10.1038/s42003-024-06086-9)
Supplement: Supplementary file 2 — Description of Additional Supplementary Files [file 42003_2024_6086_MOESM2_ESM.pdf]

Description of Additional Supplementary Files

File name 1: Supplementary Data
Description: The source data behind the graphs in the paper
